# The preparation technology and application of xylo-oligosaccharide as prebiotics in different fields: A review

**DOI:** 10.3389/fnut.2022.996811

**Published:** 2022-08-25

**Authors:** Caoxing Huang, Yuxin Yu, Zheng Li, Bowen Yan, Wenhui Pei, Hao Wu

**Affiliations:** ^1^Co-Innovation Center for Efficient Processing and Utilization of Forest Resources, Department of Bioengineering, Nanjing Forestry University, Nanjing, China; ^2^The Affiliated Zhongda Hospital of Southeast University Medical School, Nanjing, China; ^3^Department of Biomedical Engineering, School of Biomedical Engineering and Informatics, Nanjing Medical University, Nanjing, China

**Keywords:** xylo-oligosaccharide, prebiotics, bio-application, extraction, isolation

## Abstract

Xylo-oligosaccharide (XOS) is a class of functional oligosaccharides that have been demonstrated with prebiotic activity over several decades. XOS has several advantages relative to other oligosaccharide molecules, such as promoting root development as a plant regulator, a sugar supplement for people, and prebiotics to promote intestinal motility utilization health. Now, the preparation and extraction process of XOS is gradually mature, which can maximize the extraction and avoid waste. To fully understand the recent preparation and application of XOS in different areas, we summarized the various technologies for obtaining XOS (including acid hydrolysis, enzymatic hydrolysis, hydrothermal pretreatment, and alkaline extraction) and current applications of XOS, including in animal feed, human food additives, and medicine. It is hoped that this review will serve as an entry point for those looking into the prebiotic field of research, and perhaps begin to dedicate their work toward this exciting classification of bio-based molecules.

## Introduction

**FIGURE 1:**
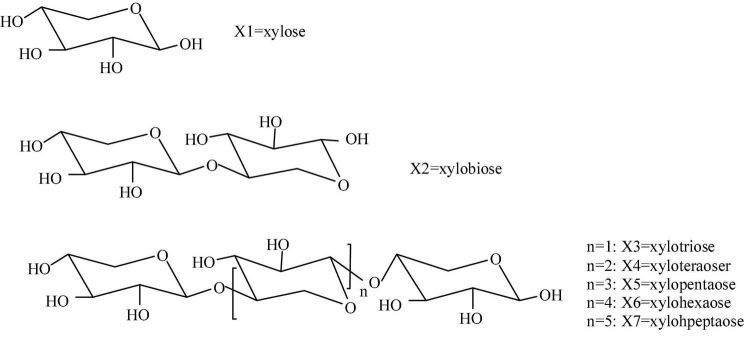
The schematic structure of xylose and xylo-oligosaccharides.

**FIGURE 2:**
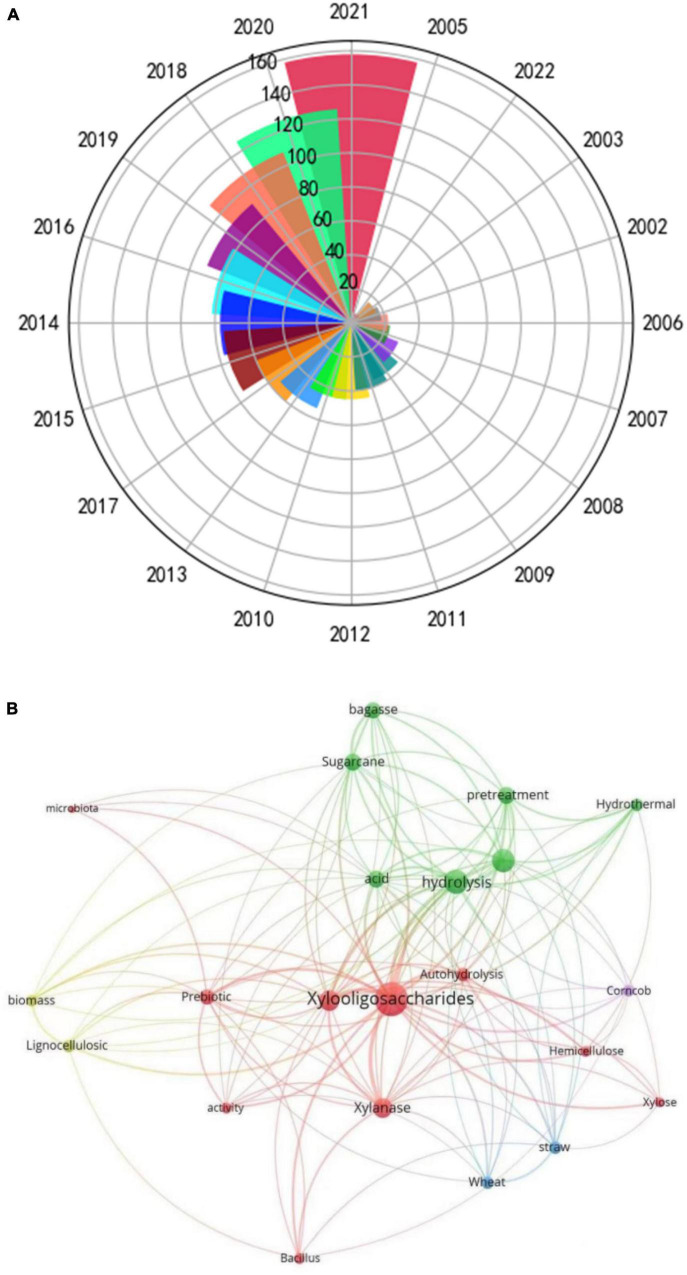
The number of published studies for xylooligosaccharides in different years **(A)**; keywords co-occurrence mapping of XOS, XOS technology, and XOS application **(B)**.

Xylo-oligosaccharide (XOS) is a type of oligosaccharide that is demonstrated to provide benefits to intestinal motility, another phrase for prebiotics (1). Chemically, XOS is the xylose-based oligosaccharides comprising 2–10 D-xylose residues linked *via* β-1,4-glycosidic bonds (as shown in Figure 1). When XOS is ingested, it can travel directly to the large intestine, where it is selectively utilized by *bifidobacteria Lactobacillus acidophilus* and *Lactobacillus casei* for their proliferation (2, 3). Hence, XOS ultimately provides the effect of inhibiting the growth of other harmful bacteria within the intestine due to targeted growth of those which are beneficial (4). It has been reported that XOS is the most efficient among the oligosaccharide peers (such as galacto-oligosaccharides, lactulose, inulin, and fructose-oligosaccharides) in promoting bifidobacterium proliferation (5). As a reflection of the increasing interest in XOS (Figure 2A), studies on XOS as prebiotics have increased year by year in the past 20 years. This is due to widespread demonstration, both *in vivo* and *in vitro*, of the health benefits provided through human consumption of XOS. As can be seen, the keywords co-occurrence mapping of XOS, XOS technology, and XOS application in Figure 2B, XOS are being studied including raw materials, production, and application. This picture vividly conveys the connectivity, diversity, and importance of research fields.

Currently, XOS can be obtained through two pathways: (a) using glycosidase and glycosyltransferase enzymes to glycosylate and resynthesize the monosaccharides to prepare XOS (6); and (b) using the chemical, physical, or biodegradation of polysaccharides technologies to prepare XOS from biomass (7). Compared to the first technology, the second technology is a practical pathway to produce XOS, which can directly depolymerize the xylan in biomass *via* hydrothermal, steam explosion, acidic, or alkali pretreatments (8–10). These approaches can render an aqueous stream that contains varying contents of XOS, which is dependent upon the hemicellulose composition of the starting materials, as well as the severity of pretreatment. There are also reports that sulfite, ionic liquid, and organic solvent pretreatments provide similar effects for different biomass to produce XOS (11). As expected, xylan-rich biomass is the ideal candidate, including straw, cottonseed shell, bagasse, and corn husks (12–14). The industrial manufacture of XOS is currently produced from corn cobs due to their high amount of hemicellulose content (∼30%) (8, 15). In addition, new technology was also proposed to prepare XOS from corn cobs. For example, Hao et al. (15) proposed delignification with hydrogen peroxide-acetic acid followed by two-step enzymatic hydrolysis to prepare XOS and monosaccharides. The results showed that the xylo-oligosaccharide yield of corn cob treated by HPAA containing 50 mM H_2_SO_4_ was 79.1%. The hemicellulose in this natural plant is mainly composed of xylan, so the hemicellulose content directly determines the yield of XOS (16).

Research for the utilization of XOS can be dated back to1960s (17). At present, the development of the oligosaccharide industry worldwide is very active, which has been formed with a multi-disciplinary research focus. With the rapid development of technology, preparation processes and areas of application for XOS continue to develop and evolve. In light of the rapid pace of development, recent and relevant works on XOS production technology (including acid hydrolysis, enzymatic hydrolysis, hydrothermal pretreatment, and alkaline extraction) and application (e.g., animal feed, food additives, and medicine) are introduced, discussed, and contextualized within this review work. We hope that this review will serve as a resource for current researchers and new entrants alike in developing their areas of research and fields of expertise.

## Technology for the preparation of xylo-oligosaccharide

**FIGURE 3:**
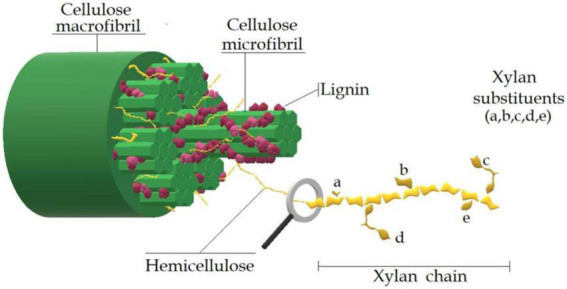
The structure of xylan in the cell wall of biomass.

Because hemicellulosic xylan is present in the cell wall of plant cells, it interacts with lignin in its natural state through covalent and physical bonds (18). As shown in Figure 3, the cellulose and hemicellulose in the cell wall of lignocellulosic biomass before pretreatment is wound around lignin, which is linked to form the lignin-carbohydrate complexes (19–21). Due to the complex structure of biomass, the xylan as polysaccharides in cell wall cannot be directly hydrolyzed by the enzyme into XOS. Hence, pretreatment for biomass or extraction of xylan from biomass can be used to produce XOS. For pretreatment, it can depolymerize and degrade the original hemicellulose to render constituent monomer sugars, oligosaccharides, and other byproducts (22, 23). Generally, the amount and structure of oligosaccharides depend on the biomass itself, the pretreatment method, and the severity of the treatment process (24). It is well-known that the existed lignin and cellulose in the cell wall can prevent the interaction between xylan and xylanase, thus reducing the formation rate and yield (25). Therefore, before enzymatic hydrolysis, the raw material must be effectively pretreated to extract xylan, and then XOS can be obtained by enzymatic hydrolysis with xylanase (26, 27). In addition, XOS can be produced by chemical degradation or pretreatment of hemicellulose in lignocellulosic materials. Now, XOS is mainly prepared by polysaccharide decomposition methods, including auto-hydrolysis, acid hydrolysis, enzyme hydrolysis, and alkali extraction (28–31). For example, XOS extracted from rice straw has been prepared by hydrothermal method and purified by gel filtration (GFC). GFC is a highly efficient purification method for the recovery of interesting XOS species and has potential applications in the pharmaceutical, food, and feed industries (32). Wheat straw was treated by hydrothermal pretreatment, alkali treatment, enzyme treatment, and their combination, which overcame the resistance structure of wheat straw and allowed selective separation into fermentable sugar and XOS (33–35). To better understand these methods, the advantages and disadvantages of these technologies were reviewed for acid hydrolysis, enzymatic hydrolysis, hydrothermal, and alkali extraction to produce XOS, which has been briefly introduced in Table 1 (9, 17, 19).

**TABLE 1:** Comparison of different methods to prepare XOS.

Materials and methods	Advantages	Disadvantages
Acid hydrolysis	1. Reduce the production of inhibitors 2. Simple process	1. Difficult to control the speed 2. High-cost input 3. High equipment requirements
Enzyme hydrolysis	1. Low production cost 2. Fewer byproducts	1. Substrate specificity 2. High price
Hydrothermal pretreatment	1. Simple process 2. Reduce equipment corrosion	1. Difficult to separate and purify 2. Low conversion of raw materials
Alkali treatment	1. Significantly improve yield 2. Good separation effect 3. Adapt to the lower temperature	1. Seriously polluted 2. High toxicity

## Acid hydrolysis of biomass for xylo-oligosaccharide production

Xylose can be prepared by partial hydrolysis of xylan with dilute acid of hydrochloric acid and sulfuric acid (36). However, this method is not often suitable for the production of XOS due to the strong acidity of the acid catalysts. High levels of severity using these acids can lead to degradation of all XOS into xylose and further degradation of xylose into furfural (37, 38). Furthermore, the use of strong acids is accompanied by expensive metallurgy requirements and corrosion inhibition regimens, adding cost to the overall process. A safer approach is for the production of XOS making use of weak and/or organic acids, such as acetic acid, butyric acid, furoic acid, and gluconic acid.

One example of a suitable acid catalyst is acetic acid. During acetic acid-catalyzed pretreatment of biomass, XOS of varying degrees of polymerization (“DP”) are found in the liquid phase among a minor quantity of xylose and furfural (10, 39). The exact profiles of the liquid solution are depended on the biomass itself and the severity of acetic acid pretreatment. Lower severities tend to favor the production of higher DP XOS, minimal xylose, and extremely minor quantities of furfural. Higher severities can still shift it in the opposite direction, even if using acetic acid in place of stronger inorganic acids. Relevant work in this subject area found that an acetic acid-based process could render XOS yields of 45.9% based on raw xylan (40). In a different work, XOS with a polymerization degree of 2–6 was prepared from poplar sawdust under the catalysis of acetic acid. The optimum reaction conditions were 170°C and 6.5% acetic acid concentration, with a pretreatment duration of 25 min. Under these conditions, the yield of xylan XOS was 36.0% (41). Due to the low purity and high color value of xylo-oligosaccharides obtained from corn straw, the acid precipitation method was used to optimize and the elution concentration of ethanol was investigated. Furthermore, XOS purity was shown to increase from 67.3 to 87.3% when the acid precipitation pH was 2 using acetic acid. Based on acid precipitation, the highest purity of XOS was 97.87% under the adsorption and elution conditions (42). Although acetic acid hydrolysis as a green technology can produce high value-added xylo-oligosaccharides from lignocellulose, about 30% of the inert xylan is still retained (43). Hence, a cascade heating process strategy can be developed for the preparation of xylo-oligosaccharides by continuous two-step acidolysis using recyclable acetic acid as a catalyst. Under the conditions of 140°C and 190°C, the yield of xylo-oligosaccharide was 63.41 and 61.44%, which were increased by 52.03 and 76.55% compared with a one-step method. In addition, the two-step cascade heating acidolysis technology can maximize the utilization of hemicellulose and cellulose components and fully realize the value-added utilization of lignocellulosic biomass (44). It is also a good method to add soft acid catalysts during acid pretreatment to prepare XOS, such as furoic acid and gluconic acid, which can achieve an XOS yield of 45.6% from sugarcane bagasse and an XOS yield of 56.2% from corncob (45, 46).

## Enzymatic hydrolysis for the production of xylo-oligosaccharide

**FIGURE 4:**
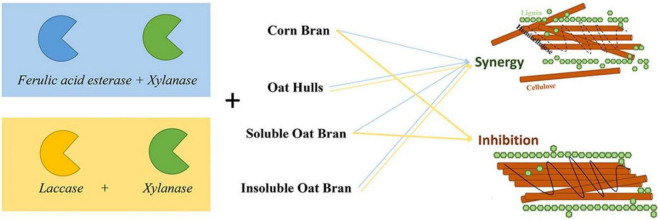
Enzyme synergy for preparing XOS is highly substrate-dependent.

Generally, many bacteria in nature can produce xylanase, which can be the medium to degrade any solid xylan into XOS (47). This method is generally used in industry. Hence, this section will review the recent work of XOS from biomass by enzymatic hydrolysis. As shown in Figure 4, there is a synergistic effect between different xylanases, and the synergistic ability of the enzyme is partly due to the increased stability of the enzyme when it is bonded to the substrates (48).

**FIGURE 5:**
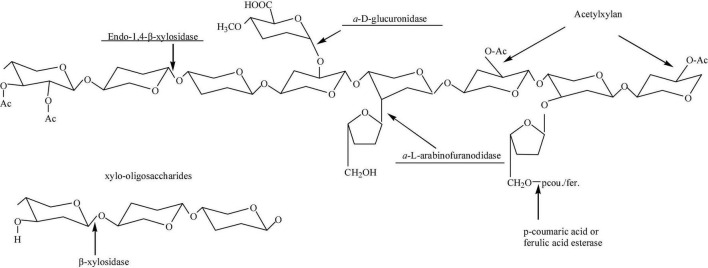
The effect of xylanase coupled with other enzymes for hemicellulose to produce XOS.

The enzymatic hydrolysis of hemicellulose with endonuclease-xylanase has fewer byproducts, which is beneficial to the isolation and purification of XOS. The work of Tuncer and Ball (49) used endo-xylanase, β-xylosidase, and α-L arabinofuranosidase to study the degradation of crude xylan of oats by xylanase. It was found that the main oligosaccharide products of oat xylan treated by endo-xylanase alone were identified as xylobiose and Xylo-pentose, and the content of xylo-tetrasaccharide was low (50, 51). Xylobiose and pentose are the main products released by endo-xylanase alone (52). The authors found that α-L arabinofuranosidase was also able to release arabinose and xylose from oat xylan (53). Guido et al. (54) studied enzymatic hydrolysis of beech xylan using xylanase without β-xylosidase. They found that about 90% (w/v) of XOS (9.18 mg/mL) could be obtained after enzymatic hydrolysis for 24 h. The obtained XOS contained 66.46% of xylose disaccharide (DP = 2), 25.10% of xylose triose (DP = 3), and a few of xylose (4.97%) (49). In addition, de Freitas et al. (55) investigated the detailed condition (xylanase load, substrate mass load, and reaction time) of enzymatic hydrolysis to prepare XOS from Aspergillus Versicolor. They found that the highest XOS yield with 60% could be achieved when the enzyme loading was 30 IU/g (54). Generally, xylan is the branched polymer with β-1,4-xylose as the main backbone and with side chains of an acetyl group, ferulic acid, uronic acid, and araban (55). To improve the degradation efficiency and purity, the supplemental enzymes of α-D-glucuronidases, acetylxylan esterases, α-L-arabinofuranosidases, ferulic acid esterases, or *p*-coumaric acid esterases should be coupled with xylanase to degrade the side chains groups during enzymatic hydrolysis process (as shown in Figure 5).

Generally, the mechanism of enzymatic hydrolysis for XOS preparation from biomass-derived is relatively clear and the yield is relatively high with specificity. Therefore, more work should be carried out to investigate how to recycle the enzyme after enzymatic hydrolysis for reuse. As the used enzyme in the enzymatic hydrolysis system still possesses a portion of catalytic ability, it can be further used for the new substance (xylan) to produce XOS.

## Hydrothermal pretreatments for the production of xylo-oligosaccharide

Hydrothermal pretreatment, also known as autohydrolysis, is a green pretreatment process whose degradation mechanism is based exclusively on the actions of hot water and pressure upon biomass (55, 56). Through liberation of acetic acid from acetate groups in the structure of hemicellulose during hydrothermal pretreatment, the soft acid catalyst is only produced *in situ*, allowing for reduced chemical cost. Generally, this method is only truly applicable to biomass with hemicellulose that bears a sufficient molar quantity of acetate groups. Therefore, this process requires optimization conditions for each biomass to be used. The chemical degradation mechanism of biomass is greatly similar to the use of soft organic acids, whereby hemicellulose is most significantly degraded into XOS, xylose, and furfural (as well as the all-important acetic acid). Hydrothermal pretreatment mainly removes hemicellulose and a small amount of lignin by degrading its sub-structures (mainly β-O-4 linkage) (57, 58). Hydrothermal pretreatment methods include liquid hot water pretreatment (auto-hydrolysis or water extraction), steam explosion pretreatment, and microwave-assisted hydrothermal pretreatment (9). The mechanism and advantages of these technologies have been briefly introduced in Table 2 (9).

**TABLE 2:** Introduction of hydrothermal pretreatment methods.

Methods	Theory	Advantages
Steam explosion	The heat of the steam and the shear forces generated by decompression decompose the lignocellulose structure	1. Rapidly heating biomass 2. Remove hemicellulose evenly
liquid hot water	At high temperatures, the H3O + generated by water will promote the cleavage of acetyl groups in hemicelluloses to form acetic acid	1. Higher quantity 2. Lower concentrations of the dissolved products
Microwave-assisted hydrothermal pretreatment	Microwave heating uses electromagnetic waves in the range of 300–300 GHz to directly coupled with dipoles in the heated material, and the energy is evenly dissipated in the entire material.	1. Reducing the generation of by-products 2. Rapid and uniform heating 3. High extraction efficiency

Mushroom culture waste liquor (WM) is rich in nutrients and hemicellulose. To improve the yield of XOS from this substrate, a suitable combination of washing and grinding pretreatment was studied, followed by intermittent and continuous flow reactors for hydrothermal treatment. The results showed that 2.3 kg of purified XOS could be produced from 30 kg of wet WM (15% dry base yield). Based on xylan mass, the theoretical yield of XOS can reach 48% (59). In another work, xylo-oligosaccharides were obtained by hydrothermal pretreatment for Eucalyptus by-product (EB) at reaction conditions of 161°C, 65 min, and a concentration of 10%. The study found that 12.4 tons of XOS could be produced from 200 tons of EB by hydrothermal pretreatment (60). In the work of Jang et al. (61), they prepare XOS from hemicellulose isolated from *Eucalyptus pellita* by liquid hot water treatment under the conditions varied based on calculated usage of combined severity factors (CSF). It is found that under the condition of CSF1.0, the maximum XOS conversion rate of 67.2% was achieved at 170°C for 50 min (62).

Generally, the obtained XOS from biomass by hydrothermal pretreatment possess fractions with higher DP (over 6). It is well-known that XOS with DP of 2–3 is the more effective prebiotic to induce intestinal bacteria (61). Hence, a combination of hydrothermal pretreatment and enzymatic hydrolysis has been proposed for biomass to produce the XOS with the content of higher-value fractions of X2-X3. For example, Fang et al. (63) used a green biorefinery process based on hot water pretreatment and enzymatic hydrolysis to achieve efficient co-production of XOS and glucose from birch sawdust. It is found that the maximum yield of XOS was 46.1% by these combined technologies (64). In addition, Su et al. (65) studied the production process of poplar XOS by combining hydrothermal pretreatment with post-hydrolysis by xylanase endo-nuclide. They found that the structural characteristics of low DP xylan indicate that XOS yield can be effectively increased by enzymatic hydrolysis with xylanase upon the liquid hydrolysate. The yield of XOS and the proportion of xylo-disaccharide (X2) to xylotriose (X3) increased from 35.4 and 46.9% to 44.6 and 78.7%, respectively (63).

Overall, the advantages of this method are a fast reaction rate, no chemical reagent, and less pollution to the environment. The mild pH conditions in hydrothermal pretreatment lead to fewer corrosion problems, avoiding the steps of waste management and acid recovery, and the degradation of polysaccharides, which provides good selectivity and the operation process is simple (65). The disadvantages are heat resistance and pressure resistance, lignin and a large number of monosaccharide residues, cumbersome separation and purification process, and low raw material utilization rate and conversion rate.

## Alkaline extraction

Alkaline extraction of lignocellulose principle is to destroy cell wall and reduces the crystallinity of cellulose by degrading hemicellulose and lignin enough so that some of their fragments are rendered alkali-soluble. This leaves a cellulose fraction with higher purity that can more easily be processed (66). Under alkali pretreatment, hemicellulose and lignin polymers can be effectively co-extracted. The extracted xylan can be further hydrolyzed by the enzyme that produces XOS. Even though some lignin fractions may still exist in the produced XOS, it is not necessary to remove lignin from the products when it is used as the additive in animal feed. It has been reported that lignin possesses a good antioxidant ability, which is a benefit for the health of the animal by scavenging reactive oxygen species (67–70).

For example, Samanta et al. (71) used corn cob to evaluate the extraction efficiency of xylan using sodium hydroxide and comparing it with potassium hydroxide. Results show that sodium hydroxide is better than potassium hydroxide in ensuring the recovery of xylan. The dry weight yield of xylan was 32.4% of original hemicellulose after being treated with 12% NaOH and steam. In addition, alkali-extracted xylan does not contain glucose or reduce sugar (72). The obtained xylan can be used as stock to produce XOS by enzymatic hydrolysis. In addition, alkali extraction can also be used in lignin-free materials to produce XOS. It is reported that XOS could be prepared from bleached kraft pulp from *Eucalyptus globulus*. Under the condition of 100°C, the maximum conversion rate of xylan to XOS in 30 min is 30.2% (71). However, Samanta et al. (73) extracted XOS from pigeon pea stems by sodium hydroxide (12%) plus steam which resulted in the recovery of XOS by up to 96%, which contained XOS, xylo-disaccharide, and xylotriose (74). Overall, alkali extraction has the advantages of a good separation effect and higher yield, but it will cause pollution and high toxicity to the environment.

## Application of xylo-oligosaccharide in different fields

Xylo-oligosaccharide is an important prebiotic, which can selectively stimulate the growth and activity of one or several bacteria to produce beneficial effects on the host, thereby improving the health of the host (73). Now, XOS is a new type of non-digestive oligosaccharides (NDOs) widely used as a functional food ingredient or supplement in Japan and China. In Japan, XOS is approved as a food ingredient under the Designation of Food for Designated Health Use (FOSHU) (75). According to the study conducted by Grand View Research Inc., the global prebiotics market was 581.0 kilotons in 2013 and is expected to reach 1,084 kilotons by 2020, growing at a CAGR of 9.3% from 2014 to 2020, and the global prebiotics market is expected to reach USD 5.75 billion by 2020. The Asia-Pacific region is expected to be the fastest-growing market by volume, growing at a CAGR of 9.6% from 2014 to 2020 (4). The increased market for XOS is due to its wide application in different fields. Hence, the application of XOS as prebiotics in the fields of animal feed, human food additives, and medicine was reviewed in this section.

## Application of xylo-oligosaccharide as animal feed

Xylo-oligosaccharide as a feed additive can enhance animal immunity, improve animal production performance, and has the characteristics of low dosage, no pollution, and no residue. Hence, XOS as the feed additive has a broad application prospect for animals (76). Many experimental results show that XOS can improve the nutrient absorption rate and feed conversion rate of animals. Added benefits include improvements to the balance of intestinal flora, enhanced immunity, as well as other functions that has also been found for XOS as animal feed (77–79). XOS alone or mixed with inulin has an obvious prebiotic effect. This was demonstrated by changes in the composition of the colon flora and its metabolic activity in animals. For example, Samanta et al. (80) studied the effects of XOS on blood biochemical characteristics of broilers. They found that the addition of 0.5% corn husk-derived XOS to broiler diets caused blood biochemical changes (81). Ding et al. (82) found that the addition of XOS in the diet of laying hens showed benefits for its intestinal health, which could enhance the number of bifidobacterium and cecal butyric acid content in the cecum of laying hens (80). In the work of Wang et al. (83), they reported that the supplementation of XOS (100 mg/kg) for chicks could increase the villus height (VH) of all intestine segments, jejunal goblet cell numbers, and the mRNA expression of zonula occludens-1 and occludin of the jejunum (82). In addition, dietary XOS supplementation can overall be described as improving cecal microflora composition and blood biochemical indices of broilers. These results further illustrate the regulatory effect of prebiotics XOS on the intestinal microflora of broilers (81). It is found that prebiotic fibers reduced pro-inflammatory responses, reduced lipopolysaccharide (LPS) translocation, and improved the secretion of IgA (S-IgA) into the lumen. In addition, xylanase has also been added to poultry and pig feeds to counter the anti-nutritional effects of non-starch polysaccharides (NSP) in normal feeds, which can provide an opportunity to improve the energy contribution of fiber and produce XOS in distal intestinal fermentation (83).

## Application of xylo-oligosaccharide as human food additives

With the development of XOS research, it was found that XOS had good characteristics, such as biological effects, and the low daily intake supplement in the food industry (25). XOS can stimulate the growth of intestinal bifidobacteria when it was added to infant milk powder, juices, yogurts, and carbonated drinks (4, 84). It can have a synergistic effect on the whole intestine and promote the maturation of the intestinal barrier of the human body (85). High purity XOS products contain at least 70–95% fraction, which is the general purity requirement for XOS used in the food industry (86). Beyond the benefits listed above, XOS has also been shown to inhibit the growth of intestinal saprophytes, accelerate intestinal peristalsis, and promote the rapid discharge of carcinogenic substances. In addition, healthy volunteers ate a balanced diet rich in XOS, which has shown that it possesses anti-inflammatory potential activity (87). For example, it was found that XOS induced the production of pro-inflammatory cytokines TNF-α, IL-1 β, IL-6, and NO in unstimulated macrophages and inhibited their production in a dose-dependent manner in lipopolysaccharide (LPS) stimulated macrophages (88). These effects may explain the immunomodulatory activity of XOS, which could enhance innate immunity and prevent cardiovascular and chronic inflammatory diseases among their other benefits (89). Yang et al. (90) combined transcriptome and metabolic analysis of XOS utilization and metabolism in adolescent *Bifidobacterium adolescentis* 15703. Compared with xylose, XOS significantly promoted the growth and fermentation performance of *B. adolescentis* 15703 (91).

Except for the prebiotic property of XOS, it can also improve the flavor, physical properties, and shelf life of food. Compared with XOS, XOS is 0.25 times sweeter than the 5% sucrose solution. When the sweetness concentration is 5% sucrose, XOS has a sour and corn silk taste (90). This low sweetness characteristic has been used in food formulations to replace sucrose. In addition, the low caloric density makes oligosaccharides to be used as expansion agents in food formulations. Because of their high moisturizing ability, they are used as humectants without increasing water activity in XOS-rich yogurts to see if they had nutritional properties and acceptable sensory properties (92–94). The physicochemical and sensory properties of XOS-concentrated yogurt at different storage times were measured. Maintaining the sensory properties of all experimental yogurts, the results indicated that the yogurts are supplemented with 3.5% XOS successful treatment in terms of overall acceptability, allowing the use of XOS to enhance the health benefits associated with these non-digestible oligosaccharides (NDO). When XOS was added to cookies, baking characteristics such as caramel flavor, darker color, crispness, and better sweetness are increased and the overall taste strength of cookies (95). It is found that the addition of 5–10% XOS in the cookies could enrich it in highly acceptable terms of physicochemical properties of diameter, height, and slightly darker color than the control (96, 97).

## Application of xylo-oligosaccharide as medicine

Xylo-oligosaccharide has the function of proliferating beneficial bacteria and can prevent and treat diarrhea and constipation (98). At the same time, XOS can absorb toxic substances and pathogenic bacteria in the intestinal tract, activate the immune system, and improve the body’s resistance to diseases, such as reducing the incidence of obesity and metabolic disorders, preventing the wide range of diseases related to oncology and cardiovascular and endocrine systems (88, 99). Hence, XOS has great prospects of development in human medicine with a dose of 0.12 g/kg body weight in adults. In addition, XOS acts as prebiotics to improve calcium and magnesium absorption, increase bone density, reduce cancer risk, reduce cardiovascular disease, and improve the immune system (100).

Several works have shown that XOS can have cytotoxic effects, leading to a reduction in the activity of acute lymphocytic leukemia cell line (101, 102). XOS was also evaluated in premature aging in clinical trials of skin health in juices containing XOS. After 8 weeks of juice-XOS intervention, the average levels of skin brightness, moisture, elasticity, pigmentation, UV, and brown spots increased by 2.7, 11, 5.1, 3.1, 6.2, and 0.6% (103). The xylo-oligosaccharides prepared from Melia xylan have a potential anti-proliferation effect when used as medicine. It could inhibit the growth of human colorectal cancer (HT-29) cells but did not affect mouse fibroblasts, confirming its biocompatibility (104). XOS, as an ideal additive for conventional drugs, has been used for infants, pregnant women, the elderly, as well as other special needs groups of patients with hypertension, hyperlipidemia, and diabetes (105). Overall, the application of XOS as medicine is based on its prebiotic property. Hence, when XOS is utilized by the patient, it may alleviate disease for the patient by regulating gut microbial flora.

**FIGURE 6:**
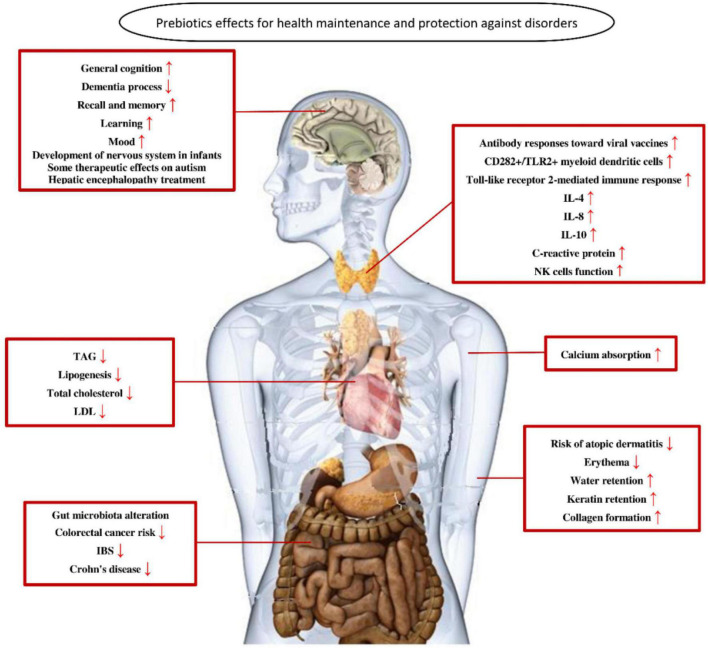
The role of probiotics in maintaining health and preventing disorders of the patient.

As can be seen in Figure 6, XOS as probiotics can maintain health and prevent disorders of the patient (106). Overall, the application of XOS as medicine is based on its prebiotic property. Hence, when XOS is utilized by the patient, it may alleviate disease for the patient by regulating gut microbial flora.

## Conclusion and prospect

Xylo-oligosaccharide has diversified effects for its wide application in different fields. In addition to providing useful modification of the physicochemical properties of foods, it also has a variety of physiological functions to improve the intestinal microflora based on the selective proliferation of bifidobacteria and stimulate mineral absorption. With the increasing research depth of XOS, people found that XOS has many advantages, such as green environmental protection, safety, non-toxic, low pollution, and the utilization rate gradually increased. XOS is widely used to improve the growth efficiency of human and animal health. In future, research into XOS should focus on its mechanism of action, applicable objects, and safe dosages, all among other issues. In addition, the advantages in the extraction and processing of XOS and resources should be further investigated. With the deepening of understanding and the decrease in production cost, XOS will usher in a new stage of development and inject new vitality into all aspects.

## Author contributions

YY and ZL conducted the literature search and wrote the first draft of the manuscript. WP and BY drafted the figures in the manuscript. HW revised the manuscript. CH rewrote the manuscript according to the reviewers. All authors have read and agreed to the published version of the manuscript.

## References

[1] Guarino MPL Altomare A Emerenziani S Di Rosa C Ribolsi M Balestrieri P Mechanisms of action of prebiotics and their effects on gastro-intestinal disorders in adults. *Nutrients.* (2020) 12:1037. 10.3390/nu12041037 32283802 PMC7231265

[2] Pandey K Naik S Vakil B. Probiotics, prebiotics and synbiotics-a review. *J Food Sci Technol.* (2015) 52:7577–87. 10.3390/ijms21249737 26604335 PMC4648921

[3] Tuohy KM Rouzaud GCM Bruck WM Gibson GR. Modulation of the human gut microflora towards improved health using prebiotics-assessment of efficacy. *Curr Pharm Design.* (2005) 11:75–90. 10.2174/1381612053382331 15638753

[4] Gupta PK Agrawal P Hegde P Shankarnarayan N Vidyashree S Singh SA Xylooligosaccharide-a valuable material from waste to taste: a review. *J Environ Res Dev.* (2016) 10:555.

[5] Macfarlane GT Steed H Macfarlane S. Bacterial metabolism and health-related effects of galacto-oligosaccharides and other prebiotics. *J Appl Microbiol.* (2008) 104:305–44. 10.1111/j.1365-2672.2007.03520.x 18215222

[6] Liu H Nidetzky B. Leloir glycosyltransferases enabled to flow synthesis: continuous production of the natural C-glycoside nothofagin. *Biotechnol Bioeng.* (2021) 118:4402–13. 10.1002/bit.27908 34355386 PMC9291316

[7] Barreteau H Delattre C Michaud P. Production of oligosaccharides as promising new food additive generation. *Food Technol Biotechnol.* (2006) 44:323–33.

[8] Carvalho AFA de Oliva Neto P Da Silva DF Pastore G. M.Xylo-oligosaccharides from lignocellulosic materials: chemical structure, health benefits and production by chemical and enzymatic hydrolysis. *Food Res Int.* (2013) 51:75–85. 10.1016/j.foodres.2012.11.021

[9] Yue P Hu Y Tian R Bian J Peng F. Hydrothermal pretreatment for the production of oligosaccharides: a review. *Bioresour Technol.* (2022) 343:126075. 10.1016/j.biortech.2021.126075 34606922

[10] Yin X Cai T Liu C Ma Y Hu J Jiang J A novel solvothermal biorefinery for production of lignocellulosic xylooligosaccharides, fermentable sugars and lignin nano-particles in biphasic system. *Carbohydr Polym.* (2022) 295:119901. 10.1016/j.carbpol.2022.119901 35988988

[11] Rezania S Oryani B Cho J Talaiekhozani A Sabbagh F Hashemi B Different pretreatment technologies of lignocellulosic biomass for bioethanol production: an overview. *Energy.* (2020) 199:117457. 10.1016/j.energy.2020.117457

[12] Khat-Udomkiri N Sivamaruthi BS Sirilun S Lailerd N Peerajan S Chaiyasut C. Optimization of alkaline pretreatment and enzymatic hydrolysis for the extraction of xylooligosaccharide from rice husk. *AMB Express.* (2018) 8:1–10. 10.1186/s13568-018-0645-9 30014174 PMC6047951

[13] Ahmad N Zakaria MR Mohd Yusoff MZ Fujimoto S Inoue H Ariffin H Subcritical water-carbon dioxide pretreatment of oil palm mesocarp fiber for xylooligosaccharide and glucose production. *Molecules.* (2018) 23:1310. 10.3390/molecules23061310 29848973 PMC6100371

[14] Dai L Gu Y Xu J Guo J Jiang K Zhou X Toward green production of xylooligosaccharides and glucose from sorghum straw biowaste by sequential acidic and enzymatic hydrolysis. *Ind Crop Prod.* (2022) 179:114662. 10.1016/j.indcrop.2022.114662

[15] Hao X Xu F Zhang J. Effect of pretreatments on production of xylooligosaccharides and monosaccharides from corncob by a two-step hydrolysis. *Carbohydr Polym.* (2022) 285:119217. 10.1016/j.carbpol.2022.119217 35287848

[16] Tunc MS van Heiningen AR. Hydrothermal dissolution of mixed southern hardwoods. *Holzforschung.* (2008) 62:539–45. 10.1515/hf.2008.100

[17] Samanta AK Jayapal N Jayaram C Roy S Kolte AP Senani S Xylooligosaccharides as prebiotics from agricultural by-products: production and applications. *Bioact Carbohydr Diet Fibre.* (2015) 5:62–71. 10.1016/j.bcdf.2014.12.003

[18] Gao Y Lipton AS Wittmer Y Murray DT Mortimer JC. A grass-specific cellulose–xylan interaction dominates in sorghum secondary cell walls. *Nat Commun.* (2020) 11:1–10. 10.1038/s41467-020-19837-z 33247125 PMC7695714

[19] Santibáñez L Henríquez C Corro-Tejeda R Bernal S Armijo B Salazar O. Xylooligosaccharides from lignocellulosic biomass: a comprehensive review. *Carbohydr Polym.* (2021) 251:117118. 10.1016/j.carbpol.2020.117118 33142653

[20] Zheng L Yu P Zhang Y Wang P Yan W Guo B Evaluating the bio-application of biomacromolecule of lignin-carbohydrate complexes (LCC) from wheat straw in bone metabolism via ROS scavenging. *Int J Biol Macromol.* (2021) 176:13–25. 10.1016/j.ijbiomac.2021.01.103 33482216

[21] Pei W Chen ZS Chan HYE Zheng L Liang C Huang C. Isolation and identification of a novel anti-protein aggregation activity of lignin-carbohydrate complex from *Chionanthus retusus* leaves. *Front Bioeng Biotechnol.* (2020) 8:573991. 10.3389/fbioe.2020.573991 33102457 PMC7546364

[22] Kumar A Singh D Chandel AK Sharma KK. Technological advancements in sustainable production of second generation ethanol development: an appraisal and future directions. In: Chandel A Sukumaran R editors. *Sustainable Biofuels Development in India.* Cham: Springer (2017). p. 299–336. 10.1007/978-3-319-50219-9_14

[23] Sun D Lv ZW Rao J Tian R Sun SN Peng F. Review of hydrothermal pretreatment on the dissolution and structural evolution of hemicelluloses and lignin. *Carbohydr Polym.* (2021) 281:119050. 10.1016/j.carbpol.2021.119050 35074121

[24] Qaseem MF Shaheen H Wu AM. Cell wall hemicellulose for sustainable industrial utilization. *Renew Sustain Energ Rev.* (2021) 144:110996. 10.1016/j.rser.2021.110996

[25] Baker JT Duarte ME Holanda DM Kim SW. Friend or foe? Impacts of dietary xylans, xylooligosaccharides, and xylanases on intestinal health and growth performance of monogastric animals. *Animals.* (2021) 11:609. 10.3390/ani11030609 33652614 PMC7996850

[26] Collins T Gerday C Feller G. Xylanases, xylanase families and extremophilic xylanases. *FEMS Microbiol Rev.* (2005) 29:3–23. 10.1016/j.femsre.2004.06.005 15652973

[27] Shen R Li HQ Zhang J Xu J. Effects of impurities in alkali-extracted xylan on its enzymatic hydrolysis to produce xylo-oligosaccharides. *Appl Biochem Biotechnol.* (2016) 179:740–52. 10.1007/s12010-016-2028-5 26922729

[28] Hao X Wen P Wang J Wang J You J Zhang J. Production of xylooligosaccharides and monosaccharides from hydrogen peroxide-acetic acid-pretreated poplar by two-step enzymatic hydrolysis. *Bioresour Technol.* (2020) 297:122349. 10.1016/j.biortech.2019.122349 31708384

[29] Chen X Li H Sun S Cao X Sun R. Co-production of oligosaccharides and fermentable sugar from wheat straw by hydrothermal pretreatment combined with alkaline ethanol extraction. *Indust Crops Prod.* (2018) 111:78–85. 10.1016/j.indcrop.2017.10.014

[30] Cano ME García-Martin A Comendador Morales P Wojtusik M Santos VE Kovensky J Production of oligosaccharides from agrofood wastes. *Fermentation.* (2020) 6:31. 10.3390/fermentation6010031

[31] Wang ZK Huang C Zhong JL Wang Y Tang L Li B Valorization of Chinese hickory shell as novel sources for the efficient production of xylooligosaccharides. *Biotechnol Biofuels.* (2021) 14:1–13. 10.1186/s13068-021-02076-9 34838122 PMC8626943

[32] Silveira MHL Chandel AK Vanelli BA Sacilotto KS Cardoso EB. Production of hemicellulosic sugars from sugarcane bagasse via steam explosion employing industrially feasible conditions: pilot scale study. *Bioresour Technol Rep.* (2018) 3:138–46. 10.1016/j.biteb.2018.07.011

[33] Moniz P Pereira H Duarte LC Carvalheiro F. Hydrothermal production and gel filtration purification of xylo-oligosaccharides from rice straw. *Ind Crop Prod.* (2014) 62:460–5. 10.1016/j.indcrop.2014.09.020

[34] Precup G Venus J Heiermann M Schneider R Pop ID Vodnar DC. Chemical and enzymatic synthesis of biobased xylo-oligosaccharides and fermentable sugars from wheat straw for food applications. *Polymers.* (2022) 14:1336. 10.3390/polym14071336 35406211 PMC9003230

[35] Attoumbré J Lesur D Giordanengo P Baltora-Rosset S. Preparative separation of glycoalkaloids α-solanine and α-chaconine by centrifugal partition chromatography. *J Chromatogr B.* (2012) 908:150–4. 10.1016/j.jchromb.2012.09.025 23040988

[36] Chen MH Rajan K Carrier DJ Singh V. Separation of xylose oligomers from autohydrolyzed *Miscanthus× giganteus* using centrifugal partition chromatography. *Food Bioprod Process.* (2015) 95:125–32. 10.1016/j.fbp.2015.04.006

[37] Huang K Luo J Cao R Su Y Xu Y. Enhanced xylooligosaccharides yields and enzymatic hydrolyzability of cellulose using acetic acid catalysis of poplar sawdust. *J Wood Chem Technol.* (2018) 38:371–84. 10.1080/02773813.2018.1500608

[38] Cebreiros F Risso F Cagno M Cabrera MN Rochón E Jauregui G Enhanced production of butanol and xylosaccharides from *Eucalyptus grandis* wood using steam explosion in a semi-continuous pre-pilot reactor. *Fuel.* (2021) 290:119818. 10.1016/j.fuel.2020.119818

[39] Yan Y Zhang C Lin Q Wang X Cheng B Li H Microwave-assisted oxalic acid pretreatment for the enhancing of enzyme hydrolysis in the production of xylose and arabinose from bagasse. *Molecules.* (2018) 23:862. 10.3390/molecules23040862 29642578 PMC6017411

[40] Bian J Peng P Peng F Xiao X Xu F Sun RC. Microwave-assisted acid hydrolysis to produce xylooligosaccharides from sugarcane bagasse hemicelluloses. *Food Chem.* (2014) 156:7–13. 10.1016/j.foodchem.2014.01.112 24629931

[41] Peng F Peng P Xu F Sun RC. Fractional purification and bioconversion of hemicelluloses. *Biotechnol Adv.* (2012) 30:879–903. 10.1016/j.biotechadv.2012.01.018 22306329

[42] Zhang H Xu Y Yu S. Co-production of functional xylooligosaccharides and fermentable sugars from corncob with effective acetic acid prehydrolysis. *Bioresour Technol.* (2017) 234:343–9. 10.1016/j.biortech.2017.02.094 28340439

[43] Wang YH Zhang J Qu YS Li HQ. Removal of chromophore in enzymatic hydrolysis by acid precipitation to improve the quality of xylo-oligosaccharides from corn stalk. *Bioresour Technol.* (2018) 249:751–7. 10.1016/j.biortech.2017.08.068 29101893

[44] Guo J Cao R Huang K Xu Y. Comparison of selective acidolysis of xylan and enzymatic hydrolysability of cellulose in various lignocellulosic materials by a novel xylonic acid catalysis method. *Bioresour Technol.* (2020) 304:122943. 10.1016/j.biortech.2020.122943 32086033

[45] Guo J Gu Y Zhou X Xu B Wang H Xu Y. Cascade temperature-arising strategy for xylo-oligosaccharide production from lignocellulosic biomass with acetic acid catalyst recycling operation. *Renew Energ.* (2021) 175:625–37. 10.1016/j.renene.2021.05.066

[46] Han J Cao R Zhou X Xu Y. An integrated biorefinery process for adding values to corncob in co-production of xylooligosaccharides and glucose starting from pretreatment with gluconic acid. *Bioresour Technol.* (2020) 307:123200. 10.1016/j.biortech.2020.123200 32222689

[47] Dai L Huang T Jiang K Zhou X Xu Y. A novel recyclable furoic acid-assisted pretreatment for sugarcane bagasse biorefinery in co-production of xylooligosaccharides and glucoase. *Biotechnol Biofuels.* (2021) 14:35. 10.1186/s13068-021-01884-3 33531058 PMC7856728

[48] Kumar V Verma D Satyanarayana T. Extremophilic bacterial xylanases: production, characteristics and applications. *Curr Biotechnol.* (2013) 2:380–99.

[49] Tuncer M Ball AS. Co-operative actions and degradation analysis of purified xylan-degrading enzymes from *Thermomonospora fusca* BD25 on oat-spelt xylan. *J Appl Microbiol.* (2003) 94:1030–5. 10.1046/j.1365-2672.2003.01943.x 12752811

[50] Zhang Q Wan G Li M Jiang H Wang S Min D. Impact of bagasse lignin-carbohydrate complexes structural changes on cellulase adsorption behavior. *Int J Biol Macromol.* (2020) 162:236–45. 10.1016/j.ijbiomac.2020.06.084 32535209

[51] Bhalla A Bischoff KM Uppugundla N Balan V Sani RK. Novel thermostable endo-xylanase cloned and expressed from bacterium *Geobacillus* sp. WSUCF1. *Bioresour Technol.* (2014) 165:314–8. 10.1016/j.biortech.2014.03.112 24725385

[52] Shahid S Tajwar R Akhtar MW. A novel trifunctional, family GH10 enzyme from *Acidothermus cellulolyticus* 11B, exhibiting endo-xylanase, arabinofuranosidase and acetyl xylan esterase activities. *Extremophiles.* (2018) 22:109–19. 10.1007/s00792-017-0981-8 29170828

[53] Juturu V Wu JC. Microbial exo-xylanases: a mini review. *Appl Biochem Biotechnol.* (2014) 174:81–92. 10.1007/s12010-014-1042-8 25080375

[54] Guido ES Silveira JT Kalil SJ. Enzymatic production of xylooligosaccharides from beechwood xylan: effect of xylanase preparation on carbohydrate profile of the hydrolysates. *Int Food Res J.* (2019) 26:713–21.

[55] de Freitas C Terrone CC Masarin F Carmona EC Brienzo M. In vitro study of the effect of xylooligosaccharides obtained from banana pseudostem xylan by enzymatic hydrolysis on probiotic bacteria. *Biocatal Agric Biotechnol.* (2021) 33:101973. 10.1016/j.bcab.2021.101973

[56] Saha BC. Hemicellulose bioconversion. *J Ind Microbiol Biotechnol.* (2003) 30:279–91. 10.1007/s10295-003-0049-x 12698321

[57] Gu Y Hu Y Huang C Lai C Ling Z Yong Q. Co-production of amino acid-rich xylooligosaccharide and single-cell protein from paper mulberry by autohydrolysis and fermentation technologies. *Biotechnol Biofuels Bioprod.* (2022) 15:1–10. 10.1186/s13068-021-02095-6 35418087 PMC8746646

[58] Li M Yi L Bin L Zhang Q Song J Jiang H Comparison of nonproductive adsorption of cellulase onto lignin isolated from pretreated lignocellulose. *Cellulose.* (2020) 27:7911–27. 10.1007/s10570-020-03357-6

[59] Huang C Jiang X Shen X Hu J Tang W Wu X Lignin-enzyme interaction: a roadblock for efficient enzymatic hydrolysis of lignocellulosics. *Renew Sustain Energ Rev.* (2022) 154:111822. 10.1016/j.rser.2021.111822

[60] Sato N Shinji K Mizuno M Nozaki K Suzuki M Makishima S Improvement in the productivity of xylooligosaccharides from waste medium after mushroom cultivation by hydrothermal treatment with suitable pretreatment. *Bioresour Technol.* (2010) 101:6006–11. 10.1016/j.biortech.2010.03.032 20378335

[61] Jang SK Kim JH Choi JH Cho SM Kim JC Kim H Evaluation of xylooligosaccharides production for a specific degree of polymerization by liquid hot water treatment of tropical hardwood. *Foods.* (2021) 10:463. 10.3390/foods10020463 33672511 PMC7923788

[62] Neto FSPP Roldán IUM Galán JPM Monti R de Oliveira SC Masarin F. Model-based optimization of xylooligosaccharides production by hydrothermal pretreatment of *Eucalyptus* by-product. *Ind Crop Prod.* (2020) 154:112707. 10.1016/j.indcrop.2020.112707

[63] Fang L Su Y Wang P Lai C Huang C Ling Z Co-production of xylooligosaccharides and glucose from birch sawdust by hot water pretreatment and enzymatic hydrolysis. *Bioresour Technol.* (2022) 348:126795. 10.1016/j.biortech.2022.126795 35121099

[64] Yan B Huang C Lai C Ling Z Yong Q. Production of prebiotic xylooligosaccharides from industrial-derived xylan residue by organic acid treatment. *Carbohydr Polym.* (2022) 292:119641. 10.1016/j.carbpol.2022.119641 35725201

[65] Su Y Fang L Wang P Lai C Huang C Ling Z Efficient production of xylooligosaccharides rich in xylobiose and xylotriose from poplar by hydrothermal pretreatment coupled with post-enzymatic hydrolysis. *Bioresour Technol.* (2021) 342:125955. 10.1016/j.biortech.2021.125955 34547709

[66] Cardenas-Toro FP Alcazar-Alay SC Forster-Carneiro T Meireles MAA. Obtaining oligo-and monosaccharides from agroindustrial and agricultural residues using hydrothermal treatments. *Food Public Health.* (2014) 4:123–39. 10.9523/j.fph.20140403.08

[67] Jackson MG. The alkali treatment of straws. *Anim Feed Sci Technol.* (1977) 2:105–30. 10.1016/0377-8401(77)90013-x

[68] Liu W Liu K Du H Zheng T Zhang N Xu T Cellulose nanopaper: fabrication, functionalization, and applications. *Nano Micro Lett.* (2022) 14:104. 10.1007/s40820-022-00849-x 35416525 PMC9008119

[69] Pei W Deng J Wang P Wang X Zheng L Zhang Y Sustainable lignin and lignin-derived compounds as potential therapeutic agents for degenerative orthopaedic diseases: a systemic review. *Int J Biol Macromol.* (2022) 212:547–60. 10.1016/j.ijbiomqc.2022.05.152 35643155

[70] Dong H Zheng L Yu P Jiang Q Wu Y Huang C Characterization and application of lignin–carbohydrate complexes from lignocellulosic materials as antioxidants for scavenging in vitro and in vivo reactive oxygen species. *ACS Sustain Chem Eng.* (2019) 8:256–66. 10.1021/acssuschemeng.9b05290

[71] Samanta AK Senani S Kolte AP Sridhar M Sampath KT Jayapal N Production and in vitro evaluation of xylooligosaccharides generated from corn cobs. *Food Bioprod Process.* (2012) 90:466–74. 10.1016/j.fbp.2011.11.001

[72] Huang C Wang X Liang C Jiang X Yang G Xu J A sustainable process for procuring biologically active fractions of high-purity xylooligosaccharides and water-soluble lignin from moso bamboo prehydrolyzate. *Biotechnol Biofuels.* (2019) 12:1–13. 10.1186/s13068-019-1527-3 31384296 PMC6661736

[73] Samanta AK Jayapal N Kolte AP Senani S Sridhar M Mishra S Application of pigeon pea (*Cajanus cajan*) stalks as raw material for xylooligosaccharides production. *Appl Biochem Biotechnol.* (2013) 169:2392–404. 10.1007/s12010-013-0151-0 23456278

[74] Henriques P Martinho M de Lurdes Serrano M de Sousa APM Alves AMB. Xylooligosaccharides production by acid hydrolysis of an alkaline extraction filtrate from *Eucalyptus globulus* bleached kraft pulp. *Ind Crop Prod.* (2021) 159:113066. 10.1016/j.indcrop.2020.113066

[75] Hijová E Bertková I Štofilová J. Dietary fibre as prebiotics in nutrition. *Cent Eur J Public Health.* (2019) 27:251–5. 10.21101/cejph.a5313 31580563

[76] Penksza P Juhász R Szabó-Nótin B Sipos L. Xylo-oligosaccharides as texture modifier compounds in aqueous media and in combination with food thickeners. *Food Sci Nutr.* (2020) 8:3023–30. 10.1002/fsn3.1177 32724566 PMC7382132

[77] Liu X Lin Q Yan Y Peng F Sun R Ren J. Hemicellulose from plant biomass in medical and pharmaceutical application: a critical review. *Curr Med Chem.* (2019) 26:2430–55. 10.2174/0929867324666170705113657 28685685

[78] Li DD Ding XM Zhang KY Bai SP Wang JP Zeng QF Effects of dietary xylooligosaccharides on the performance, egg quality, nutrient digestibility and plasma parameters of laying hens. *Anim Feed Sci Technol.* (2017) 225:20–6. 10.1016/j.anifeedsci.2016.12.010

[79] Zhou J Wu S Qi G Fu Y Wang W Zhang H Dietary supplemental xylooligosaccharide modulates nutrient digestibility, intestinal morphology, and gut microbiota in laying hens. *Anim Nutr.* (2021) 7:152–62. 10.1016/j.aninu.2020.05.010 33997343 PMC8110867

[80] Samanta AK Kotte AP Elangovan AV Dhali A Senani S Sridhar M Effects of corn husks derived xylo-oligosaccharides on performance of broiler chicken. *Indian J Anim Sci.* (2017) 87:640–3.

[81] Ribeiro T Cardoso V Ferreira LMA Lordelo MMS Coelho E Moreira ASP Xylo-oligosaccharides display a prebiotic activity when used to supplement wheat or corn-based diets for broilers. *Poult Sci.* (2018) 97:4330–41. 10.3382/ps/pey336 30101299

[82] Ding XM Li DD Bai SP Wang JP Zeng QF Su ZW Effect of dietary xylooligosaccharides on intestinal characteristics, gut microbiota, cecal short-chain fatty acids, and plasma immune parameters of laying hens. *Poult Sci.* (2018) 97:874–81. 10.3382/ps/pex372 29294100

[83] Wang Q Wang XF Xing T Li JL Zhu XD Zhang L The combined impact of xylo-oligosaccharides and gamma-irradiated *Astragalus* polysaccharides on growth performance and intestinal mucosal barrier function of broilers. *Poult Sci.* (2021) 100:100909. 10.1016/j.psj.2020.11.075 33518329 PMC7936216

[84] Vazquez MJ Alonso JL Domınguez H Parajo JC. Xylooligosaccharides: manufacture and applications. *Trends Food Sci Technol.* (2000) 11:387–93. 10.1016/s0924-2244(01)00031-0

[85] Wu YB Lin KW. Influences of xylooligosaccharides on the quality of Chinese-style meatball (kung-wan). *Meat Sci.* (2011) 88:575–9. 10.1016/j.meatsci.2011.02.018 21388751

[86] Aachary AA Prapulla SG. Xylooligosaccharides (XOS) as an emerging prebiotic: microbial synthesis, utilization, structural characterization, bioactive properties, and applications. *Compr Rev Food Sci F.* (2011) 10:2–16. 10.1111/j.1541-4337.2010.00135.x

[87] Lian Z Zhang Q Xu Y Zhou X Jiang K. Biorefinery cascade processing for converting corncob to xylooligosaccharides and glucose by maleic acid pretreatment. *Appl Biochem Biotechnol.* (2022) 256:1–13. 10.1007/s12010-022-03985-7 35674923

[88] Lecerf JM Dépeint F Clerc E Dugenet Y Niamba CN Rhazi L. Xylo-oligosaccharide (XOS) in combination with inulin modulates both the intestinal environment and immune status in healthy subjects, while XOS alone only shows prebiotic properties. *Br J Nutr.* (2012) 108:1847–58. 10.1017/s0007114511007252 22264499

[89] Aziz M Jacob A Matsuda A Wu R Zhou M Dong W Pre-treatment of recombinant mouse MFG-E8 downregulates LPS-induced TNF-α production in macrophages via STAT3-mediated SOCS3 activation. *PLoS One.* (2011) 6:e27685. 10.1371/journal.pone.0027685 22114683 PMC3217009

[90] Yang J Tang Q Xu L Li Z Ma Y Yao D. Combining of transcriptome and metabolome analyses for understanding the utilization and metabolic pathways of xylo-oligosaccharide in *Bifidobacterium adolescentis* ATCC 15703. *Food Sci Nutr.* (2019) 7:3480–93. 10.1002/fsn3.1194 31762999 PMC6848847

[91] Chehimi M Vidal H Eljaafari A. Pathogenic role of IL-17-producing immune cells in obesity, and related inflammatory diseases. *J Clin Med.* (2017) 6:68. 10.3390/jcm6070068 28708082 PMC5532576

[92] Kim MJ Yoo SH Jung S Park MK Hong JH. Relative sweetness, sweetness quality, and temporal profile of xylooligosaccharides and luo han guo (*Siraitia grosvenorii*) extract. *Food Sci Biotechnol.* (2015) 24:965–73. 10.1007/s10068-015-0124-x

[93] Patel S Goyal A. Functional oligosaccharides: production, properties and applications. *World J Microbiol Biotechnol.* (2011) 27:1119–28. 10.1007/s11274-010-0558-5

[94] Raman R Raguram S Venkataraman G Paulson JC Sasisekharan R. Glycomics: an integrated systems approach to structure-function relationships of glycans. *Nat Methods.* (2005) 2:817–24. 10.1038/nmeth807 16278650

[95] Mumtaz S Rehman SU Huma N Jamil A Nawaz H. Xylooligosaccharide enriched yoghurt: physicochemical and sensory evaluation. *Pak J Nutr.* (2008) 7:566–9. 10.3923/pjn.2008.566.569

[96] Juhász R Penksza P Sipos L. Effect of xylo-oligosaccharides (XOS) addition on technological and sensory attributes of cookies. *Food Sci Nutr.* (2020) 8:5452–60. 10.1002/fsn3.1802 33133548 PMC7590280

[97] Pareyt B Finnie SM Putseys JA Delcour JA. Lipids in bread making: sources, interactions, and impact on bread quality. *J Cereal Sci.* (2011) 54:266–79. 10.1016/j.jcs.2011.08.011

[98] Ayyappan P Abirami A Anbuvahini NA Tamil Kumaran PS Naresh M Malathi D Physicochemical properties of cookies enriched with xylooligosaccharides. *Food Sci Technol Int.* (2016) 22:420–8. 10.1177/10820132115617567 26644158

[99] Yuan L Li W Huo Q Du C Wang Z Yi B Effects of xylo-oligosaccharide and flavomycin on the immune function of broiler chickens. *PeerJ.* (2018) 6:e4435. 10.7717/peerj.4435 29527412 PMC5842763

[100] Yan F Tian S Chen H Gao S Dong X Du K. Advances in xylooligosaccharides from grain byproducts: extraction and prebiotic effects. *Grain Oil Sci Technol.* (2022) 5:98–106. 10.1016/j.gaost.2022.02.002

[101] Ashwini A Ramya HN Ramkumar C Reddy KR Kulkarni RV Abinaya V Reactive mechanism and the applications of bioactive prebiotics for human health. *J Microbiol Methods.* (2019) 159:128–37. 10.1016/j.mimet.2019.02.019 30826441

[102] Ando H Ohba H Sakaki T Takamine K Kamino Y Moriwaki S Hot-compressed-water decomposed products from bamboo manifest a selective cytotoxicity against acute lymphoblastic leukemia cells. *Toxicol InVitro.* (2004) 18:765–71. 10.1016/j.tiv.2004.03.011 15465641

[103] Kaprelyants L Zhurlova O Shpirko T Pozhitkova L. Xylooligosaccharides from agricultural by-products: characterisation, production and physiological effects. *Харчова наука і технологія* (2017) 11:25–34. 10.15673/fst.v11i3.606

[104] Chang HC Liang CH Lin YJ Lin YH Lin YH Kuan CM. Investigation of the synergistic effect of berry juice and xylooligosaccharides on skin health: a clinical evaluation. *J Food Nutr Res.* (2020) 8:268–72. 10.12691/jfnr-8-6-4

[105] Sharma K Morla S Khaire KC Thakur A Moholkar VS Kumar S Extraction, characterization of xylan from *Azadirachta indica* (neem) sawdust and production of antiproliferative xylooligosaccharides. *Int J Biol Macromol.* (2020) 163:1897–907. 10.1016/j.ijbiomac.2020.09.086 32946939

[106] Valdemiro Carlos S. The importance of prebiotics in functional foods and clinical practice. *Food Nutr Sci.* (2011) 2:133–44. 10.4236/fns.2011.22019

